# Molecular characterization of a Tax-2C protein variant identified in Brazilian subjects infected by HTLV-2C

**DOI:** 10.1186/1742-4690-11-S1-P110

**Published:** 2014-01-07

**Authors:** Adele Caterino-de-Araujo, Mariana Cavalheiro Magri, Umberto Bertazzoni, Maria Grazia Romanelli

**Affiliations:** 1 Centro de Imunologia, Instituto Adolfo Lutz, Secretaria de Estado da Saúde de São Paulo, São Paulo, SP., Brasil; 2 Laboratório de Investigações Médicas em Hepatologia por Vírus (LIM-47), Faculdade de Medicina, Universidade de São Paulo, São Paulo, SP., Brasil; 3 Department of Life and Reproduction Sciences, Section of Biology and Genetics, University of Verona, Verona, Italy

## 

Although the etiologic difference in pathogenic properties of HTLV-1 and HTLV-2 still remains unclear, it has been suggested that it could be attributed to the differential structure and activities of their transactivating Tax proteins. Tax-1 and Tax-2, although having 85% amino acid (aa) similarity, present phenotypic differences consistent with a more robust transformation capacity of Tax-1. Interestingly, the HTLV-2C Brazilian variant present in Amerindians and in IDU from urban areas is genotypically close to HTLV-2A but Tax-2C possesses an additional 25aa in the C-terminal region similar to that of Tax-2B. We have already demonstrated that Tax-1 and Tax-2B have several common domains, but present differential cellular distribution. To add some information concerning the structure and site domains present in Tax-2C we conducted the present study. We have obtained the Tax-2C sequence from 25 different HTLV-2C subjects and analyzed the aminoacid homology between Tax-2B and Tax-2C variants. We found that they differ for amino acidic substitutions in eleven different positions that may affect cellular localization or post-translational modification. Studies on phenotypic properties and cellular localization of Tax-2C are in progress. Support: MCT/CNPq # 303545/2012-7, CAPES, IAL # 49D/2010), Brazil.

